# Infrared single photon detector based on optical up-converter at 1550 nm

**DOI:** 10.1038/s41598-017-15613-0

**Published:** 2017-11-10

**Authors:** Peng Bai, Y. H. Zhang, W. Z. Shen

**Affiliations:** 1 Key Laboratory of Artificial Structures and Quantum Control, Department of Physics and Astronomy, Shanghai Jiao Tong University, Shanghai, 200240 China; 2 Collaborative Innovation Center of Advanced Microstructures, Nanjing, 210093 People’s Republic of China

## Abstract

High performance single photon detector at the wavelength of 1550 nm has drawn wide attention and achieved vast improvement due to its significant application in quantum information, quantum key distribution, as well as cosmology. A novel infrared up-conversion single photon detector (USPD) at 1550 nm was proposed to work in free-running regime based on the InGaAs/ InP photodetector (PD)- GaAs/AlGaAs LED up-converter and Si single photon avalanche diode (SPAD). In contrast to conventional In_0.53_Ga_0.47_As SPAD, the USPD can suppress dark count rate and afterpulsing efficiently without sacrificing the photon detection efficiency (PDE). A high PDE of ~45% can be achieved by optical adhesive coupling between up-converter and Si SPAD. Using a developed analytical model we gave a noise equivalent power of 1.39 × 10^−18^ WHz^1/2^ at 200 K for the USPD, which is better than that of InGaAs SPAD. This work provides a new single photon detection scheme for telecom band.

## Introduction

Single photon detector working at the wavelength of 1550 nm is an essential device for quantum information in optical fiber communication system^1–6^, which has attracted more and more attention nowadays. Currently available single photon detectors at this wavelength mainly include superconducting detectors, single photon avalanche diode (SPAD) and frequency up-conversion based detectors.

After years of effort, superconducting nanowire single photon detector (SNSPD) is close to all of the specifications of an ideal single photon detector, which have a high system detection efficiency (>90%), low dark count rate (<1 cps without any background radiation), low time jitter (~150 ps), and short reset time (~40 ns)^7^. But the operation temperature (<3 K) of the SNSPD is the biggest stumbling block for large-scale application. Another single photon detector is up-conversion detector working by means of sum-frequency generation in nonlinear optical crystal (periodically poled lithium niobate (PPLN)), which can convert telecom-band photons into near infrared photons to be detected by a commercial Si avalanche photodiode (APD)^8,9^. This kind of up-conversion single photon detector has high photon detection efficiency (PDE), high counting rate and has no afterpulsing problem under strong pump field. But the dark count rate of this detector can also reach up to ~10^5^ cps^8,10^. Recent progress of this detector achieved the dark count rate 20–100 cps corresponding to the PDE around 5–25% with a weak pump power^11^. However, the relatively complex optical setup and narrow spectral sensitivity prevent this kind of single photon detectors from using in most applications.

InGaAs SPAD driven in Geiger mode has obvious advantage in quantum key distribution system application owing to its high compatibility with existing optical fiber telecommunication system, high operation temperature (~200 K with Peltier cooling), compact size and relatively lower cost. Unfortunately, it suffers from the high dark count rate due to the tunnel-assisted generation of carriers in InGaAs absorption layer^12^. So InGaAs SPAD is usually designed and known as SAGM^13^ (separate absorption, grading, and multiplication) to suppress the dark count rate by means of reducing the electrical field of absorption layer. Nevertheless, the dark count rate is still around 1000 cps. Moreover, the high density of deep level center in InP multiplication layer cause a sever afterpulsing, which makes the InGaAs SPAD have to operate in gate-mode. Recent research on gate-mode InGaAs SPAD achieved 55% photon detection efficiency, which approaches the performance limit of APD^14^. It is disappointed that severe afterpulsing probability (>10%) still can’t be avoided. Besides, the uncertainty of the expected arrival time of the photons requires the InGaAs SPAD to operate in free running regime instead of gate-mode in many tasks^15^. However, the detection efficiency of the free running InGaAs SPAD based on negative feedback avalanche photodiode is only around 10% and the afterpulseing (>2%) is still can’t be suppressed efficiently^15,16^.

A potential attempt is that one can set InGaAs as the absorption layer and Si as the multiplication layer. Compared with InP, Si multiplication layer has higher ionization ratio of electron and hole, lower defect density, as well as less defect level, which lead to lower dark count rate(<100 cps) and lower afterpulsing probability(<1%) with a high photon detection efficiency(>70%) for Si avalanche photodiodes (APDs). However, the lattice mismatch between Si and InGaAs makes it impossible to fabricate the device by directly epitaxial growth. It is reported that using wafer bonding technique, InGaAs/Si p-i-n detector^17,18^ and InGaAs/Si avalanche diodes^19^ have been successfully fabricated. But the performance of the device still needs to be optimized which can’t meet the practical requirement of high-performance single photon detector.

Throughout years of development, semiconductor up-conversion technique has attracted tremendous research effort and made great progress in pixelless imaging^20–23^. This kind of up-conversion device can work under room temperature with weak excitation source condition. Mature fabrication technology and compact structure of this device make it promising for large-scale application. Besides, the high internal up-conversion quantum efficiency^24^ (~80%) of the up-converter offers new thinking and method to fabricate a high performance single photon detector at the wavelength of 1550 nm.

In this work, we proposed a novel single photon detector called semiconductor infrared up-conversion single photon detector (USPD) working in free-running regime at the wavelength of 1550 nm. The USPD consists of a PD-LED up-converter and a Si SPAD. Due to the excellent performance of both PD-LED up-converter and Si SPAD, the overall USPD could acquire a high PDE. At the same time, utilization of Si instead of InP as the multiplication layer can suppress the dark count and afterpulsing efficiently. Considering different integrating method between PD-LED up-converter and Si SPAD, we systematically investigated the PDE and NEP of the USPD and give the performance limit of USPD device. Moreover, a comparative work was presented among state-of-the-arts single photon detectors at the communication band to discuss the superiority of the USPD.

## Device model

### Principle and superiority of the USPD

**Figure 1:**
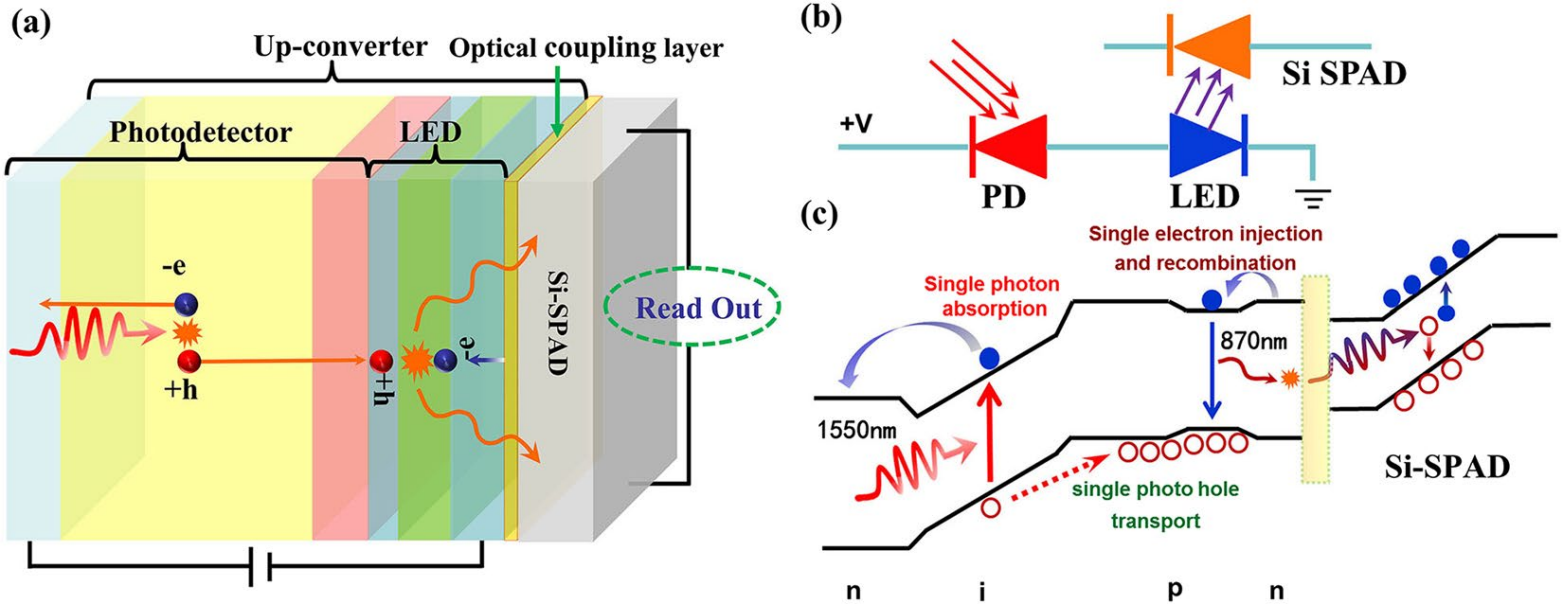
Device model schematic diagram of USPD (**a**) Structure of the USPD device, (**b**) equivalent circuit diagram of the USPD, (**c**) band diagrams of USPD.

The schematic structure of USPD is shown in Fig. 1(a). The USPD is composed of an up-converter and a Si SPAD, which could be coupled in three different ways. The up-converter consists of an InGaAs PD and a GaAs LED integrated by wafer fusion. The InGaAs PD is known as the traditional p-i-n structure, which is reverse biased. The LED is AlGaAs/GaAs/AlGaAs double heterostructure and is forward biased. When the USPD operating, photons at the wavelength of 1550 nm were absorbed by InGaAs PD first and transformed into electrical current (photon-generated carriers) under reverse bias. The photon-generated carriers migrating to the active layer of the LED recombine and luminesce at the wavelength of 870 nm. The emitted near infrared photons can be detected by a Si SPAD. The equivalent circuit diagram and band diagram of USPD are shown in Fig. 1(b,c) respectively. It is important to note that the up-conversion photons have to go through an optical coupling process to ensure more photons can pass into the Si SPAD, which will be discussed in detail in the following sections. And the extra energy for up-conversion process is from applied electric field.

The band diagram indicates the micro-mechanism of the up-conversion and single photon detection process. It is noted that USPD could make use of the high absorption of InGaAs PD at 1550 nm and the excellent performance of Si SPAD simultaneously. One great advantage of USPD is that it can suppress the dark count and afterpulsing effectively, which is mainly attribute to the relative separation between absorption zone and multiplication zone. The photon is absorbed in InGaAs layer of PD, meanwhile carriers multiplication occurs in Si SPAD. These two parts could be controlled conveniently with two independent circuits due to the different working mode. Though Si SPAD has to work in Geiger-mode, which requires a reverse-bias voltage well above the breakdown voltage, PD operates in a much lower voltage. The independent voltage control makes it possible for each function part to work in the optimized condition and thus obtain good performance. The design principle of the USPD device is utilizing Si multiplication layer of the Si SPAD as the multiplication layer of USPD. Contrast to a conventional InGaAs-SPAD, Si-SPAD has a much lower dark count rate and afterpulsing effect because of high material quality of Si. Such characteristic design of USPD can suppress the afterpulsing probability to the same level as that of Si-SPAD and enable it to operate in the free-running regime without sacrificing photon detection efficiency. For the same reason, the dark count rate (DCR) of USPD is very low. The DCR of the USPD origins from two aspects. One is the built-in DCR of Si SPAD, the other comes from the dark luminescence of LED which is caused by the dark current of the up-converter. This is quite different from the case of the conventional SPAD. In a conventional SPAD, DCR is mainly determined by the material itself, the background radiation is usually suppressed significantly and the effect can be negligible. However, for the USPD, the Si SPAD is coupled with the fore up-converter and it can ‘see’ the emission from the up-converter. Even though the up-converter is not illuminated, the LED could also luminescence due to the dark current of the up-converter. Because the PD and LED in the up-converter are diode in back-to-back configuration, the total dark current of the up-converter consist of dark current from the one in the reversed bias, i.e. the dark current of PD, and background radiation photocurrent of the InGaAs PD at the same time. This is different from the case of a discrete detector, because the background radiation photocurrent of PD in the up-converter could equally result in the dark luminescence of LED. Currently, the dark current for a specially designed InGaAs PD (25 μm diameter) is ~4 fA at room temperature^25^. And the current induced by background radiation is calculated to be ~10^−5^ fA for 180°FOV at 300 K using

1 $${I}_{bg}=\int \frac{e{g}_{PD}\eta (\lambda )P(\lambda )\lambda }{hc}d\lambda =\int \frac{e{g}_{PD}\eta (\lambda )\pi cA}{{\lambda }^{5}\exp (hc/{k}_{B}T\lambda )}d\lambda $$

Where *e* is the elementary charge, *h* is the Pluck constant, *g*_*PD*_ is the gain of the InGaAs PD, *c* is the speed of light in vacuum, *T* is temperature in Kelvins. In the case of a photodiode, each detected photon contributes exactly one electron to the signal^26^. So the gain of the InGaAs PD is ${g}_{PD}=1$. It is clear that the current induced by background radiation is much smaller than the dark current of PD. Therefore, the overall dark current of the up-converter is approximately determined by the dark current of PD. In addition, both of the dark current and background current) are sensitive to temperature and can be suppressed by reducing the working temperature. For example, if the operating temperature was reduced to 200 K, both of the currents would be 4–6 orders lower than that of room temperature. In light of such a low dark current, DCR caused by dark luminescence of the up-converter is the same level as that of Si-SPAD. As a result, the DCR of the USPD is also the same level as that of the Si-SPAD. By the way, the dead time is also mainly influenced by the multiplication layer, therefore, the dead time should also be determined by that of the Si SPAD.

### Transport property of USPD

**Figure 2:**
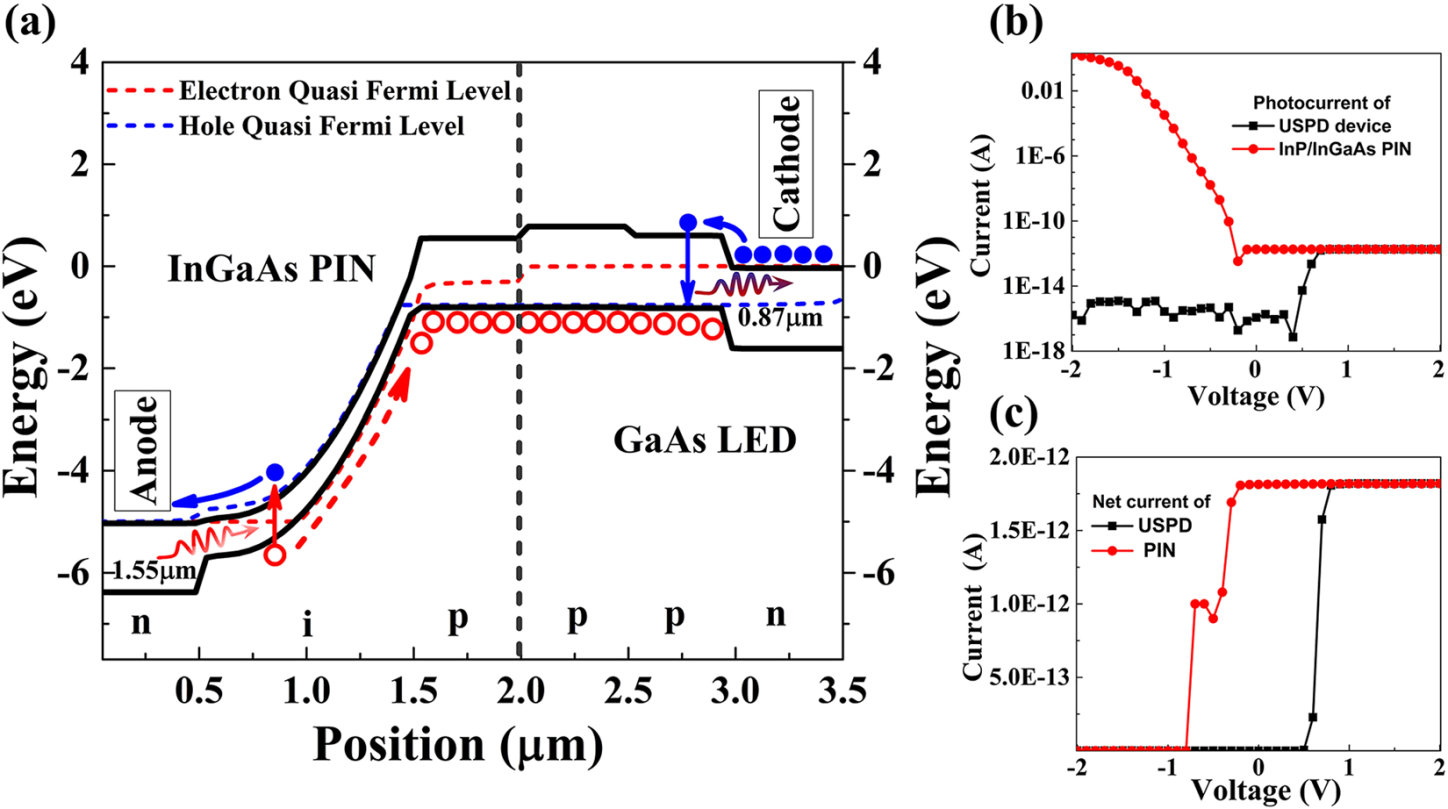
Calculated results of (**a**) band diagram of the up-converter under 5 V forward bias, (**b**) photocurrent of PIN and up-converter at different bias voltage, (**c**) net photocurrent (subtract dark current from the photocurrent) of PIN and up-converter at different bias voltages.

To better understand the working mechanism of USPD, the detailed carrier transport process need to be know firstly. Because the up-converter and Si SAPD is electric isolated, we only focus on the carrier transport process in the up-converter. The TCAD-based approach^27^ is applied to investigate the band structure and transport characteristic of the up-converter at different biases. In the calculation, we consider different recombination process, including the SRH, Auger and spontaneous recombination. Taking a conventional up-converter^21^ as an example, Fig. 2(a) presents the band diagram at a forward bias of 5 V. It is clear to see that once a photon at the wavelength of 1550 nm were absorbed by InGaAs layer, a pair of electron and hole is generated and then seperated rapidly under the electrical field of depletion region. Passing through the interface of PD and LED interface without any barrier, the hole would transport to the well of the LED smoothely. And the electron would be swept to the anode immediately. Meanwhile, to balance the charge in the up-converter, the electrons would be injected into the active layer of LED from the cathode under the positive bias of LED and recombination with hole. From this point of view, the up-converter seems like an electrical pump. To obtain a high efficiency of recombination, the active layer of the LED need to be highly p-doped. Thereby, there are always holes there ‘waiting’ for electrons to recombination, which could guarantee a good time resolution.

It should be noticed that though the photo-generated holes will travel through the wafer fusion interface, the up-conversion process doesn’t depend on the photo-generated holes. Due to the high p-doped level of both sides of the wafer bonded interface and LED active layer, the holes is the majority carriers. The concentration of the majority carriers is approximately constant at low injection level. The migration of the sparse holes generated from the weak incident light just like inject to a hole-ocean. For low-level injection, the average time it takes for a majority carrier to recombine is much longer than the minority carrier lifetime. Therefore, for low-level excitation in this situation, the majority carrier lifetime can be assumed to be infinitely long^28^. On the other hand, the electrons do not need to get through the wafer fusion interface. That is to say, the quality of the wafer bonded interface and the heterojunction between the PD and the LED would hardly affect the up-conversion.

The calculated I-V curve was show in Fig. 2(b,c). Unlike a single p-i-n photodetector, the up-converter shows a different I-V characteristic. When applied reverse bias on the up-converter, the device would not work because the PIN is forward biased and the LED is reverse biased. On the contrary, when applied forward bias on the up-converter, the PIN is reversed biased and the LED is forward biased, which is exactly accord with the operation condition of these two parts. The LED determines a turn-on behavior, the applied bias first drops across the LED part and then additional bias goes to the photodiode part. Such I-V characteristic has been confirmed in the wafer bonded up-converter in mid-infrared^29^. We can find that the photocurrent of the USPD is almost exactly the same as the single InGaAs PIN when the bias above the threshold voltage (Fig. 2(b)). The effect of the LED looks like a voltage divider. The net current (subtract dark current from photocurrent) of the USPD and single PIN turned out the same result (Fig. 2(c)).

Such I-V characteristics also indicate that the heterojunction between the PD and LED has no influence on the performance of the USPD device. Previous experimental studies also verified that the wafer bonded PD-LED up-converter would not degrade the performance of either PIN or LED^21^. And the up-conversion efficiency of the up-converter with an optimized GaAs LED shows an internal up-conversion efficiency of 76% at room temperature^22^, which reveal that a much higher up-conversion efficiency may be achieved if the PIN is also optimized. In a word, thanks to the unique up-conversion mechanism, the photo-generated holes have no harmful effect on the transport property of the USPD and it is reasonable to presume that the up-conversion efficiency is determined by the performance of the independent PD and LED.

### High photon extraction efficiency of LED

**Figure 3:**
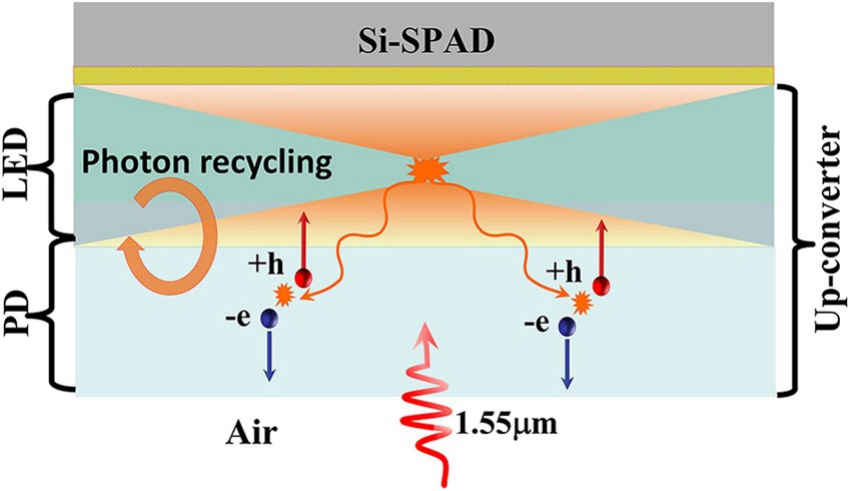
Schematic diagram of the luminescence of the LED in the up-converter and photon recycling.

To obtain high up-conversion efficiency, it is very important to increase the photon extraction efficiency of LED (external quantum efficiency of LED) as high as possible. We know that the LED emission is isotropic, which limits a photon extraction efficiency of 50% for a planar structure. However, in the USPD device, the special structure of the up-converter enables a much higher photon extraction efficiency than a single LED. Firstly, due to the reflection at the semiconductor-air interface, the escape probability of the emitted photons is only ~2% at the PD/air interface, which means 98% light will be confined in the up-converter device. Secondly, the photons back-propagating to the PD side will be reabsorbed by the InGaAs layer and reproduce photocarriers and then repeat the up conversion process, which can be regard as a ‘photon recycle process’ (shown in Fig. 3). The high absorption coefficient of the InGaAs (~6 × 10^4^ /*cm*) can ensure a sufficient reabsorption of the back-propagating photons with an appropriate layer thickness (1~2 μm).

Actually, there won’t be any photon escaping from the device at the PD side attribute to the photon recycle process. Therefor the PD in the up-converter not only works as an absorber of the 1550 nm incident light, but an ideal reflector for the 870 nm photons emitted by the LED. Thus the only limited factor of the efficiency of the USPD is the optical coupling between the up-converter and the Si SPAD, which will be discussed in detail in the following sections.

**Figure 4:**
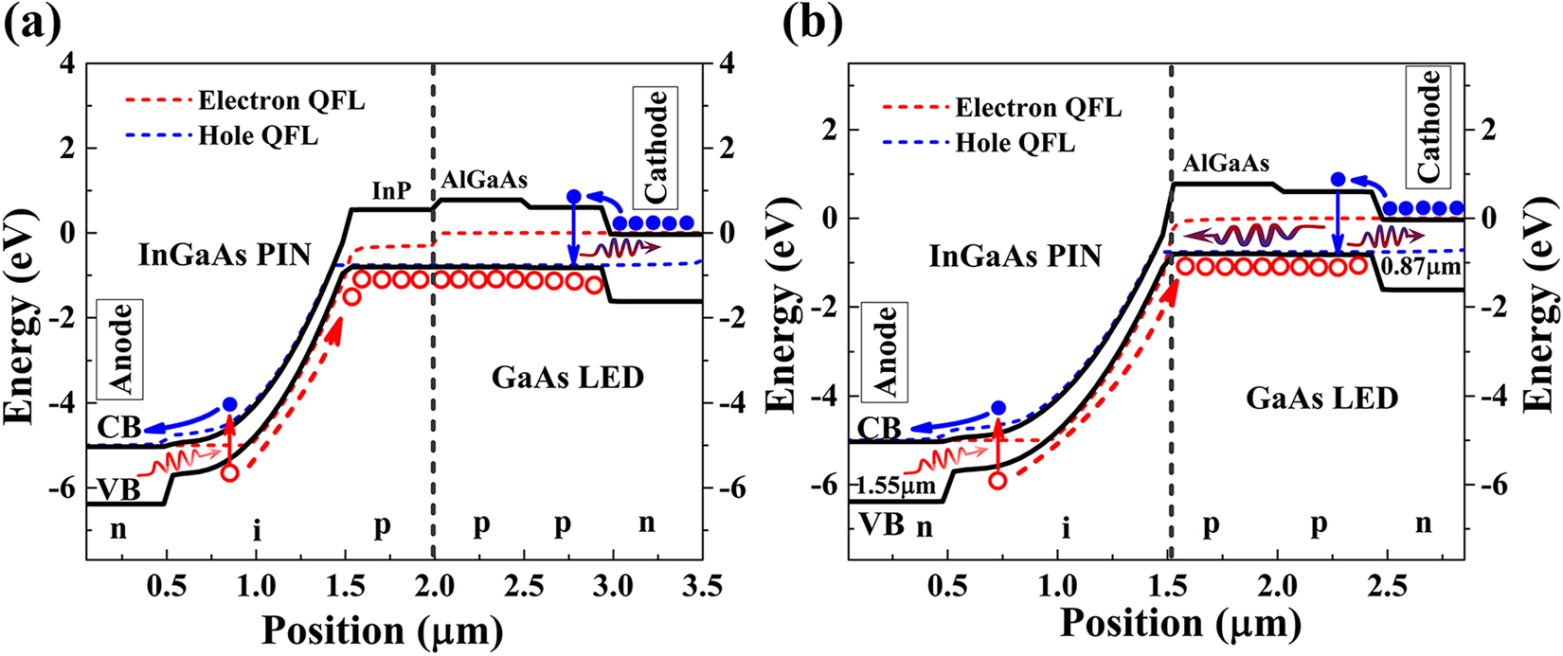
Band diagram of the up-converter (**a**) with p-InP cap layer (**b**) without p-InP cap layer.

The above analysis is obtained under the assumption that the all back-propagating photons are efficiently absorbed in the InGaAs layer. In fact, the p-InP cap layer of the InGaAs PIN also has a high absorption coefficient (~10^4^ /*cm*) at 870 nm, which will absorb most of the back-propagating photons but can’t generate photocarriers efficiently due to the relative low field at this region. Therefore, a better choice is to remove the p-InP cap layer before wafer fusion in practical application. And the p-AlGaAs layer at the common region can substitute the p-InP after the PIN wafer fusion. According to the band diagram of the up-converter in Fig. 4, the removal of the p-InP layer would not influence the operation of the USPD.

Considering three times of photon recycle and neglecting the time of light propagation and carriers transportation, nearly 90% (50% for the first time, 25% for the second recycle, 12.5% for the third recycle) of the photons can be extracted from the up-converter. The only problem of photon recycle may be the increase of the time jitter. Using the method introduced in the next section, the time jitter of the USPD in this situation is calculated also comparable to the Si SPAD.

## Performance evaluation of USPD

The performance of a single-photon detector should be assessed, in terms of its spectral range, detection efficiency, the noise equivalent power, dark count rate, dead time and time jitter. As discussed above, dark count rate and dead time of USPD is mainly determined by Si SPAD because of the unique design of USPD. Therefore, we focus mainly on the time jitter, PDE, noise, NEP and D^*^ to evaluate the performance of the USPD.

### Time resolution of the USPD

Time resolution (time jitter) of the single photon detector is a crucial factor which usually directly determines the performance of the detector in many applications^30^. So it is necessary to calculate the time jitter of the USPD before evaluating other performance. The time jitter of the USPD device can be given by

2 $${\rm{\Delta }}{t}=\sqrt{{{t}}_{pin}^{2}+{{t}}_{trans}^{2}+{{t}}_{spont}^{2}+{{t}}_{ph}^{2}+{{t}}_{si}^{2}}$$

Where *t*_*pin*_ is the optical response time for the InGaAs PIN, *t*_*trans*_ is the transport time of the photo-generated carriers, *t*_*spont*_ is the spontaneous lifetime of the LED, *t*_*ph*_ is the transmission time of the photons emitted by GaAs LED, *t*_*si*_ is the timing resolution of the Si SPAD.

### Photon detection efficiency

Photon detection efficiency (PDE) is an important characteristic for a single photon detector. The PDE of USPD device is determined by efficiency of PD, LED, Si-SPAD, as well as the optical coupling efficiency between LED and Si SPAD.

3 $$\begin{array}{rcl}\eta  & = & {\eta }_{PD}{\eta }_{LED}{\eta }_{Si}\\  & = & (1-R)(1-{e}^{-\alpha d}){\eta }_{PD}^{in}{\eta }_{LED}^{in}{\eta }^{couple}{\eta }_{Si}\end{array}$$

Where ${\eta }_{PD}$ and ${\eta }_{LED}$ are the external efficiency of the InGaAs PD and GaAs LED respectively, ${\eta }_{Si}$ is the PDE of the Si SPAD. *R* is the reflectivity of the surface of the InGaAs PD, *α* is the absorption coefficient of InGaAs, *d* is the thickness of InGaAs absorption layer, ${\eta }_{PD}^{in}$ is the photoelectric transformation efficiency of the InGaAs PD. ${\eta }_{LED}^{in}$ denotes the ratio of number of photons generated in the LED to the number of electron-hole pairs injected into the LED active region. ${\eta }^{couple}$ is the optical coupling efficiency between the LED and Si SPAD.

### Response of USPD

When the up-converter is not illuminated, the current is the sum of dark current ${I}_{dark}$ and photo current induced by background radiation ${I}_{bg}$. Accordingly, the near infrared emission (or dark luminescence) power of an up-converter under dark condition is

4 $${P}_{dark,UP}=\frac{{I}_{dark,PD}+{I}_{bg,PD}}{e}\frac{hc}{{\lambda }_{out}}{\eta }_{LED}$$

where ${\lambda }_{out}$ is the emitting wavelength of LED. When the up-converter is illuminated by infrared light with power density ${P}_{in}$, the up-conversion near infrared emission power produced by up-conversion is

5 $${P}_{ph,UP}={\eta }_{UP}{P}_{in}$$

where the subscript ‘UP’ denotes the up-converter, ${\eta }_{UP}$ is the external up-conversion efficiency, which is defined as the ratio between the output near infrared light power and input infrared power

6 $${\eta }_{UP}={\eta }_{PD}{\eta }_{LED}\frac{{\lambda }_{in}}{{\lambda }_{out}}$$

where ${\lambda }_{in}$ is the incident wavelength of USPD. The ultimate effective photocurrent in Si SPAD of the USPD device (UD) is

7 $${I}_{ph,UD}={\eta }_{Si}\frac{e{\lambda }_{out}}{hc}{P}_{ph,UP}$$

where the subscript ‘UD’ denotes the USPD. Using the definitions, we get the USPD device (UD) responsivity

8 $${R}_{UD}=\frac{{I}_{ph,UD}}{{P}_{in}}=\frac{e{\lambda }_{out}}{hc}{\eta }_{Si}{\eta }_{UP}$$

### Noise, NEP and D*of USPD device

In single photon detection applications, a high value of PDE is certainly desirable, but it is by no means the only practical consideration. Noise is also another key parameter to affect the performance of devices. It could thereby determine the noise equivalent power (NEP), the most widely quoted figure of merit of photodetectors^2^.

The current noise of up-converter comes from two parts, PD noise and LED noise

9 $${i}_{n,UP}={({i}_{n,PD}^{2}+{i}_{n,LED}^{2})}^{1/2}$$

The noise density of an InGaAs photovoltaic photodetector is expressed as

10 $${i}_{n,PD}^{2}={i}_{n,dark,PD}^{2}+{i}_{n,bg,PD}^{2}+{i}_{n,joh,PD}^{2}+{i}_{n,1/f,PD}^{2}$$

Here, ${i}_{n,dark,PD}={(2e{g}_{PD}{I}_{dark,PD}{\rm{\Delta }}f)}^{1/2}$ is the dark current noise, which is mainly caused by the generation recombination noise, ${\rm{\Delta }}f$ is the measurement bandwidth. ${i}_{n,bg,PD}={(2e{g}_{PD}{I}_{bg,PD}{\rm{\Delta }}f)}^{1/2}$ is the noise density produced by background radiations, ${i}_{n,joh,PD}={(\frac{4{k}_{B}T}{{R}_{D}}{\rm{\Delta }}f)}^{1/2}$ is Johnson noise existing in any resistance type device, where ${k}_{B}$ is the Boltzmann’s constant, *T* is the operating temperature of the device, *R*_*D*_ is the differential resistance of the device. It should be emphasized that both of dark noise and background noise generally exceeds the Johnson noise. And the *1/f* noise is complex and usually not the main source of the noise in InGaAs PD. So the Johnson noise and *1/f* noise is negligible in the following discuss.

As for the noise of the LED, on the one hand, since the InGaAs PD is reverse biased and the GaAs LED is forward biased when the up-converter is operating, the dark current is determined by the reverse biased diode^21^. On the other hand, the differential resistance of the InGaAs PD is much higher than LED, so the noise contribution from the LED current can be negligible^31^. So the LED noise ${i}_{n,LED}$ is determined by the current from InGaAs PD

11 $${i}_{n,LED}={(2e{I}_{dark,PD}{\rm{\Delta }}f+2e{I}_{bg,PD}{\rm{\Delta }}f)}^{1/2}$$

And the current noise of up-converter corresponding to an emission power noise

12 $${P}_{n,UP}=\frac{hc}{e{\lambda }_{out}}{\eta }_{LED}{i}_{n,UP}$$

Similarly, the noise of USPD device (UD) can also be written as

13 $${i}_{n,UD}={({i}_{n,UP,UD}^{2}+{i}_{n,Si,UD}^{2})}^{1/2}$$

where ${i}_{n,UP,UD}$ is the noise caused by fluctuation of the up-converter emission power

14 $${i}_{n,UP,UD}={\eta }_{Si}\frac{e{\lambda }_{out}}{hc}{P}_{n,UP}$$

${i}_{n,Si,UD}$ is the noise in the Si SPAD part

15 $${i}_{n,Si,UD}={(2e{I}_{ph,Si}{\rm{\Delta }}f+2e{I}_{dark,Si}{\rm{\Delta }}f+\frac{4{k}_{B}T}{{R}_{D,Si}})}^{1/2}$$

The first term of ${i}_{n,Si,UD}$ is the shot noise caused by the light incident onto the Si photodiode, the second term is the dark current noise, and the third term is the Johnson noise which can be ignored. It is known that the USPD could be looked up as a detector. Photons at the wavelength of 1550 nm are absorbed and a corresponding current signal is generated in the end. According to Eqs (8) to (14), the current noise of USPD as an overall detector should be

16 $$\begin{array}{rcl}{i}_{n,UD}^{2} & = & 2e{\rm{\Delta }}f{({\eta }_{Si}{\eta }_{LED})}^{2}({I}_{bg,PD}+{I}_{dark,PD})\\  &  & +2e{\rm{\Delta }}f{({\eta }_{Si}{\eta }_{LED})}^{2}({I}_{bg,PD}+{I}_{dark,PD})\\  &  & +2e{\rm{\Delta }}f{\eta }_{Si}{\eta }_{LED}({I}_{bg,PD}+{I}_{dark,PD})+2e{I}_{dark,Si}{g}_{Si}{\rm{\Delta }}f\end{array}$$

The first item comes from the InGaAs PD, the second item is caused by the LED, and the last two items originates from the Si SPAD. So, the NEP of the USPD device is

17 $$NEP=\frac{{i}_{n,UD}}{{R}_{UD}}=\frac{hc}{e{\lambda }_{out}}{[2e{\rm{\Delta }}f({I}_{dark,PD}+{I}_{bg,PD})(2+\frac{1}{{\eta }_{LED}{\eta }_{Si}})+\frac{2e{g}_{Si}{I}_{dark,Si}{\rm{\Delta }}f}{{({\eta }_{LED}{\eta }_{Si})}^{2}}]}^{1/2}$$

And the corresponding normalized detectivity D* is

18 $$D\ast =\frac{\sqrt{A{\rm{\Delta }}f}}{NEP}$$

It should also be noted that due to its unique design of USPD, the actual noise that the output terminal could ‘feel’ only includes the noise of Si SPAD itself and the noise resulted from photon fluctuation generated by the up-converter. This is because the USPD and Si SPAD are electrical isolation^32^, the current noise of the up-converter could not transfer to the output terminal. That means the current noise of USPD in Eq. (15) could be simplified to the last two terms

19 $$NEP=\frac{{i}_{n,UD}}{{R}_{UD}}=\frac{hc}{e{\lambda }_{out}}{[2e{\rm{\Delta }}f\frac{1}{{\eta }_{LED}{\eta }_{Si}}({I}_{dark,PD}+{I}_{bg,PD})+\frac{2e{g}_{Si}{I}_{dark,Si}{\rm{\Delta }}f}{{({\eta }_{LED}{\eta }_{Si})}^{2}}]}^{1/2}$$

And according to the estimation from Eq. (15), the noise would thereby decrease about 50%. The electrical isolation of USPD is another advantage of such kind of single photon detector, which could decrease the noise of the total detector and thereby increase the D*.

## Result and Discussion

### Time resolution

For a conventional InGaAs SPAD, the time jitter is about 50 ps^2^, which includes the optical response time for the InGaAs PIN and the response time of the corresponding circuit. In view of the high absorption of the InGaAs and the extremely low time jitter of the InGaAs SPAD, it is reasonable to set the optical response time for the InGaAs PIN itself *t*_*pin*_ ≈ 0. Considering the up-conversion process, once the incident light is absorbed by the InGaAs layer and generate carriers, the LED side will inject the same number of electrons into the active layer immediately. The generation of the carriers and the injection of electrons are almost synchronized. Thus the *t*_*trans*_ can be negligible in the time jitter calculation of the USPD. *t*_*ph*_ is the transmission time of the photons emitted by GaAs LED which is calculated in the order of femtosecond. *t*_*si*_ is the timing resolution of the Si SPAD and *t*_*si*_ ≈ 50 *ps* for a high-performance SPAD^33^. *t*_*spont*_ is the spontaneous lifetime of the LED, which is determined by the bimolecular recombination coefficient (*B*_*T*_) and the majority carrier concentration (*N*_*A*_) of the active layer of the LED (*t*_*spont*_ = (*B*_*r*_ × *N*_*A*_)^−1^). In the USPD device, the active layer is usually highly doped (~10^19^ *cm* ^−3^) for a high radiation recombination efficiency and the bimolecular recombination coefficient is *B*_*r*_ = 1.8 × 10^−8^, 1.9 × 10^−9^, 7.2 × 10^−10^ *cm* ^3^/*s* at 90 K, 185 K, 300 K^34^, corresponding to the spontaneous lifetime of the LED is 5.6 ps, 53 ps, 138 ps respectively. Consequently, the time resolution of the USPD can be estimated as 50.3 ps, 72.9 ps, 147 ps at 90 K, 185 K, 300 K respectively. Considering the photon recycle, the time jitter is about 150 ps, 219 ps, 441 ps at 90 K, 185 K, 300 K respectively, which is also acceptable and comparable to the Si SPAD.

### Optical coupling & Photon detection efficiency

Taking the photon detection efficiency given by equation (2) under consideration, the InGaAs PD is usually designed with anti-reflection coating corresponding to the *R* = 13%. If assuming all incoming 1550 nm photons were absorbed by the InGaAs intrinsic layer, we have $(1-{e}^{-\alpha d}\approx 1)$. Moreover, benefiting from mature fabrication processing, ${\eta }_{PD}^{in}$ can approach 100% when the InGaAs PD is well designed and fabricated. Meanwhile, the ${\eta }_{LED}^{in}$ also can be close to 100% under extremely low injection density through optimizing design^24^. Previous research has demonstrated that the wafer fusion process would not degrade the performance of either InGaAs PD or GaAs LED^21^. In view of all above conditions, we have reason to believe that the up-conversion internal efficiency of the PD-LED up-converter can get close to 100% if both of two devices were well designed, fabricated, and wafer fused perfectly. And the photon detection efficiency $({\eta }_{Si})$ of Si SPAD is a certain value for a specific wavelength. According to the equation (2), the PDE of the USPD depends only on the optical coupling efficiency $({\eta }^{couple})$ between the LED and Si SPAD.

The simplest way of optical coupling between up-converter and Si-SPAD is spatial optical coupling in which lens or non-spherical lens are used to focus the photons emitted from LED on the active area of the Si SPAD. Unfortunately, the optical setup makes the device not so compact and the overall PDE of the device is poor because of the low photon escape probability (<2%) at the GaAs/air interface. The external efficiency of the GaAs LED is only 1.33%. Considering 40% PDE of Si-SPAD at the wavelength of 870 nm^35^, the PDE of the USPD is only 0.463%.

To improve the PED of the USPD, an effective attempt is that we can directly integrate the up-converter with the Si SPAD by wafer fusion. According to the conventional theory of optics^24^, the ${\eta }^{couple}$ of this way can get ~24% using

20 $${\eta }^{couple}=\frac{1}{4}{(\sin {\theta }_{c})}^{2}[1-{(\frac{{n}_{GaAs}-{n}_{Si}}{{n}_{GaAs}+{n}_{Si}})}^{2}]$$

where ${\theta }_{c}$ is the critical angle at the GaAs/Si interface, ${n}_{Si},{n}_{GaAs}$ are the refractive indexes of Si and GaAs. The external efficiency of the GaAs LED is ~24% (corresponding to ${\eta }_{LED}^{in}=100 \%$). Taking the typical value of the PDE (40%) of Si-SPAD at 870 nm into consideration, the PED of the USPD is then calculated to be $\eta =8.4 \%$.

Another method is to use optical adhesive to integrate the up-converter and Si SPAD which is widely used in the bonding of fiber array. It is well known that if the refractive index of the optical adhesive is close to the GaAs (3.58) and Si (3.42), we will get a high ${\eta }^{couple}$. But there is currently no such an optical adhesive whose refractive index is higher than 3. The refractive index of most common optical adhesive is ~1.55. ${\eta }^{couple}$ is around 10% if thickness of the optical adhesive layer is thick enough. But if the thickness of the optical adhesive is comparable to the wavelength of the photon emitted by the LED, the photon tunneling effect will occur which can lead to a much higher ${\eta }^{couple}$.

Theoretical value of the ${\eta }^{couple}$ caused by photon tunneling effect can reach up to 100%. An optical coupling efficiency as high as 81% has been achieved experimentally^36^. So it is reasonable to assume an optical efficiency of 70% by integrating the up-converter and Si SPAD with optical adhesive. Using the typical value of the PDE of Si-SPAD at the wavelength of 870 nm, the PED of the USPD is then calculated as $\eta =24.4 \%$.

**Figure 5:**
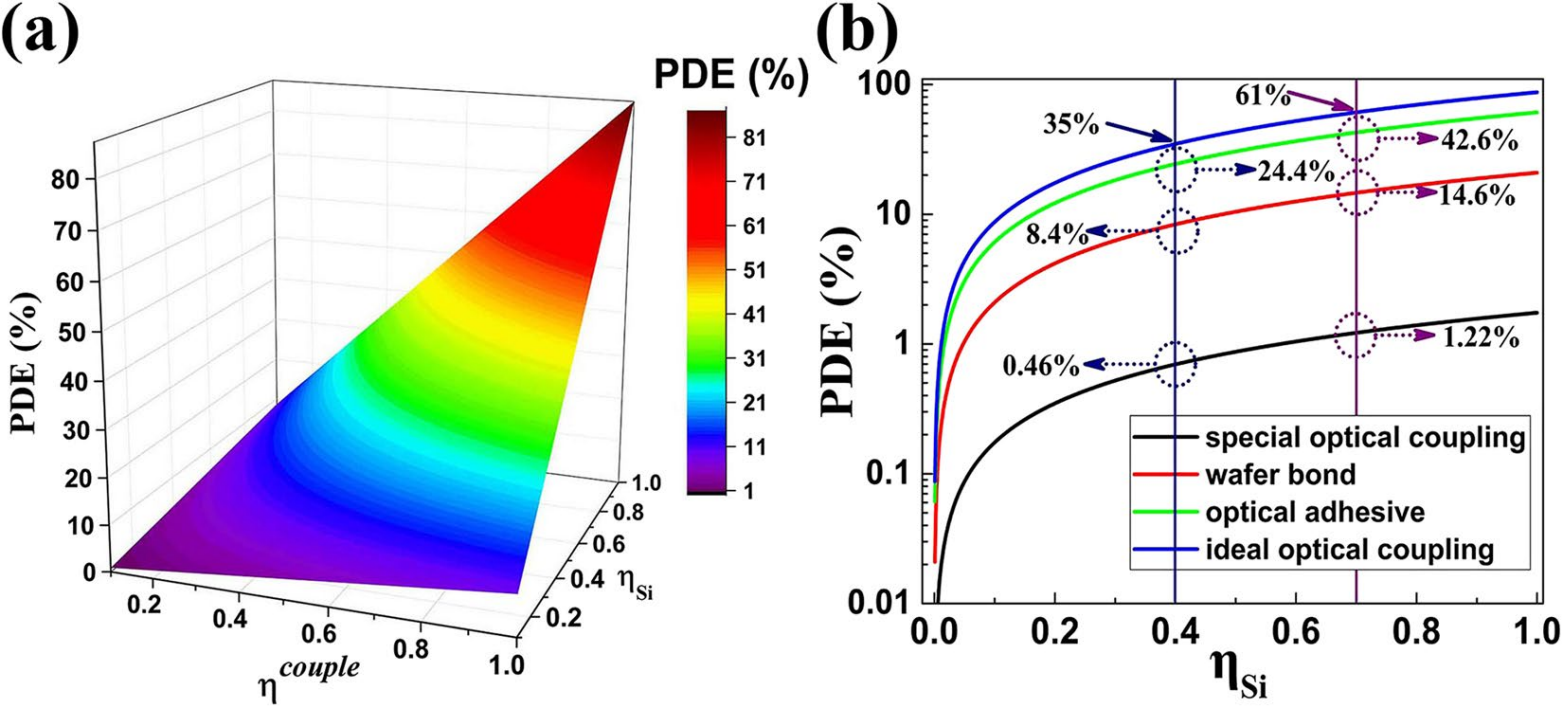
(**a**) Dependence of photon detection efficiency on optical coupling efficiency $({\eta }^{couple})$ and photon detection efficiency of Si-SPAD $({\eta }_{Si})$, (**b**) Photon detection efficiency for three different ways of optical coupling.

The photon detection efficiency for different optical coupling efficiency is shown in Fig. 5. Once the way of the optical coupling is determined, the PDE is only dependent on the photon detection efficiency of the Si SPAD. The PDE of Si SPAD ${\eta }_{Si}$ varies with the wavelength. At the present, the typical value is 40% at the wavelength of 870 nm. And the peak response of Si SPAD usually occurs at 650 nm where the PDE could be as high as 70%^35^. If LED is designed to emit at such a shorter wavelength, the USPD then will achieve a much higher PDE of 42.6%, which is comparable to the theoretical limit value of PDE for InGaAs SPAD. The theoretical limit value of PDE of USPD is also presented in blue line, which corresponds to an ideal optical efficiency (100%) of the optical adhesive. PDE is 35% and 61% when Si SPAD operates at 870 nm and 650 nm respectively.

### NEP & D*

**Figure 6:**
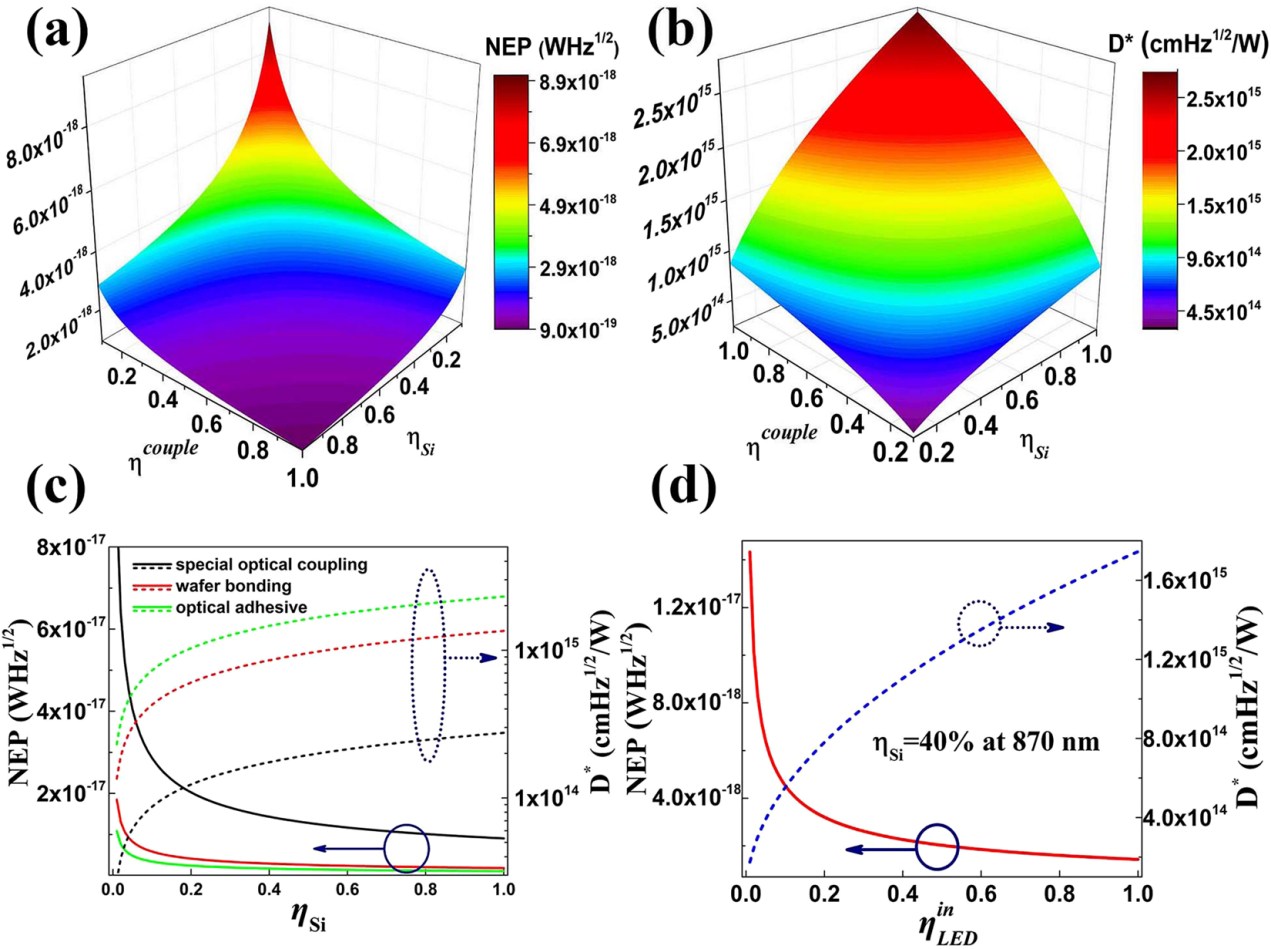
(**a**,**b**) Theoretical $NE{P}_{UD}$ and ${D}_{UD}^{\ast }$ at different optical coupling efficiency (*η* ^*couple*^) and photon detection efficiency of Si-SPAD (*η*_*si*_), (**c**) Theoretical ${D}_{UD}^{\ast }$ and $NE{P}_{UD}$ at different photon detection efficiency of Si-SPAD (*η*_*si*_) for different ways of optical coupling. Also presented is (d) the dependence of ${D}_{UD}^{\ast }$ and $NE{P}_{UD}$ on the internal efficiency of LED $({\eta }_{LED}^{in})$ at a specific PDE value of 40% for a typical commercial Si-SPAD.

The result of the calculated NEP and D^*^ of USPD device at 200 K is shown in Fig. 6. It is shown that when the ${\eta }_{Si}={\eta }^{couple}=1$, the USPD would reach its performance limit ${\rm{NEP}}={\rm{9.06}}\times {{\rm{10}}}^{-{\rm{19}}}{{\rm{WHz}}}^{1/2}$ (Fig. 6(a)) corresponding to a maximum ${{\rm{D}}}^{\ast }={\rm{2.76}}\times {{\rm{10}}}^{{\rm{15}}}{\rm{cm}}\,{{\rm{Hz}}}^{1/2}/W$(Fig. 6(b)) which is also close to the theoretical performance limit of InGaAs-SPAD $({\rm{NEP}}={\rm{1.85}}\times {{\rm{10}}}^{-{\rm{19}}}{{\rm{WHz}}}^{1/2})$. Similar to the case of PDE, once the way of the optical coupling is determined, the performance of USPD is only dependent on the photon detection efficiency of the Si SPAD (Fig. 6(c)).

When *η*_*si*_ = 40% (at 870 nm), NEP are $1.43\times {10}^{-17}{{\rm{WHz}}}^{1/2}$, $2.93\times {10}^{-18}{{\rm{WHz}}}^{1/2}$ and $1.71\times {10}^{-18}{{\rm{WHz}}}^{1/2}$ for spatial optical coupling, wafer bond and optical adhesive, respectively. Using an ideal Si-SPAD (${\eta }_{Si}=70 \%$ at 650 nm), NEP are $1.08\times {10}^{-17}{{\rm{WHz}}}^{1/2}$, $2.21\times {10}^{-18}{{\rm{WHz}}}^{1/2}$ and $1.29\times {10}^{-18}{{\rm{WHz}}}^{1/2}$ for the three different coupling ways. The corresponding D* is ${\rm{2.3}}\times {{\rm{10}}}^{{\rm{14}}}{{\rm{cmHz}}}^{1/2}/W$,${\rm{1.13}}\times {{\rm{10}}}^{{\rm{15}}}{{\rm{cmHz}}}^{1/2}/W$ and ${\rm{1.93}}\times {{\rm{10}}}^{{\rm{15}}}{{\rm{cmHz}}}^{1/2}/W$ respectively. It is noted that in some of the investigations of single photon detectors, the ideal NEP is calculated in terms of $NEP=\sqrt{2D}h\nu /\eta$, where D is the dark count rate^2^. Our results agree well with the results obtained this way.

Fig. 6(d) gives the dependence of NEP and D* on the internal efficiency of LED for the ideal optical adhesive coupling $({\eta }^{couple}=70 \% )$. The typical value of photon detection efficiency for a best performance commercial Si-SPAD is 40% at 870 nm, which lead to the performance limit for USPD is ${\rm{NEP}}={\rm{1.43}}\times {{\rm{10}}}^{-{\rm{18}}}{{\rm{WHz}}}^{1/2}$ and ${{\rm{D}}}^{\ast }={\rm{1.74}}\times {{\rm{10}}}^{{\rm{15}}}{{\rm{cmHz}}}^{1/2}/W$ with ${\eta }_{LED}^{in}=100 \% \& {\eta }^{couple}=70 \%$.

### Superiority and feasibility

**Figure 7:**
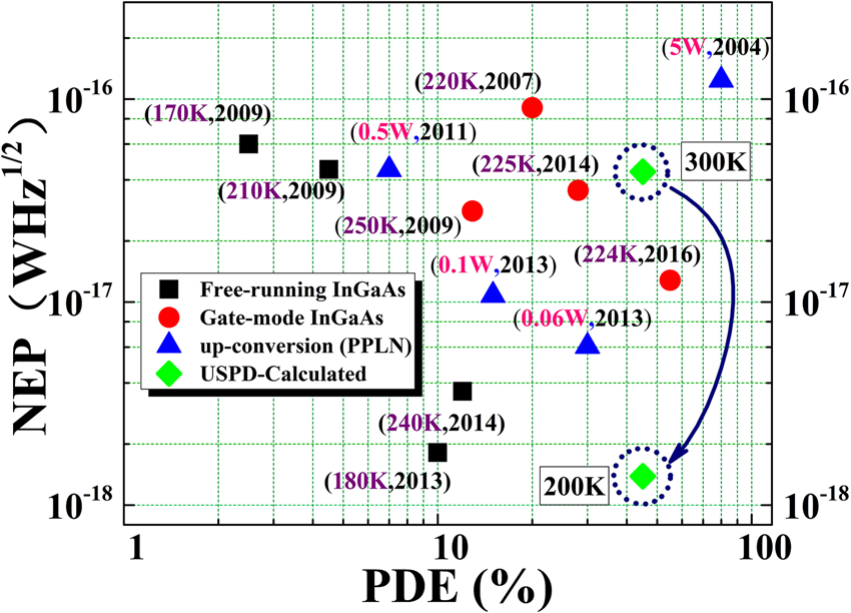
The NEP and PDE achievements for different kind of single photon detectors. Free-running InGaAs SPADs^15,39–41^ were plotted as black squares. Gate-mode InGaAs SPADs^14,42–44^ and optical up-conversion SPADs^8,11,45,46^ were shown as red circles and blue triangles respectively. The diamond in the dash circle representative the calculated results of USPD. The first item in the bracket is the operating condition and the second one in the bracket is the time of report.

In most cases, PDE is a very important parameter to characterize a single photon detector. However, NEP represents the level of signal-to-noise ratio (SNR). Lower NEP corresponds to a better SNR and high D*. The performance of a single photon detector could be characterized effectively by considering PDE and NEP (or D*) simultaneously. An ideal single-photon detector should be the one for which: the PDE and D* is as high as possible. In contrast, the dark-count rate, the dead time, is as low as possible. However, in practical application, high the PDE and D* may accompany by high dark-count rate and other performance drawbacks. Therefore, an excellent single photon detector has to be a trade-off consideration of the overall performances. Considering the fact that most of the single photon detector system is characterized with NEP, we use the parameters of PED and NEP, instead of D*, to characterize the performance. In Fig. 7, a comprehensive evaluation of several widely used single photon detectors at present is given. The first item in each bracket is the operating condition and the second one in the bracket is the reported time. A desired single photon detector should be the one that falls in the bottom right area in NEP-PDE diagram, which means high PDE, low NEP and high SNR.

Using the proposed model and the typical value^25^ of noise current for a mature commercial InGaAs PD, we get the calculated result of $NE{P}_{UD,300K}=4.39\times {10}^{-17}\,WH{z}^{1/2}$ at room temperature, and $NE{P}_{UD,200K}=1.39\times$ ${10}^{-18}\,WH{z}^{1/2}$ at 200 K for an USPD (*η*_*si*_ = 70% and *η* ^*couple*^ = 70%) with ~45% PDE. Currently, Gate-mode InGaAs SPAD (shown in Fig. 7 red circles) can achieve high PDE, but the SNR is not so good and the severe afterpulsing and dark count probability still restrict the application. And many applications, requires the SPAD to operate in the free-running regime. Free-running InGaAs SPAD (black squares) can achieve a high SNR, but the maximum PED is just around 10%. Another single photon detector is up-conversion detectors (blue triangles) working by means of sum-frequency, which is widely used in many quantum communication systems. But this kind of single photon detector still can’t simultaneously satisfy the high PDE and high SNR. It is clear that the USPD operating under thermoelectric cooling temperature (shown in Fig. 7 diamond in dash circles) has a high PDE and low NEP (high SNR). Meanwhile, significant reduction of afterpulsing also makes USPD a potential excellent single photon detector for extensive application.

Thanks to the current mature commercial Si-SPAD, the key points for fabrication of the USPD device only include fabrication of up-converter and realization of efficient optical coupling between up-converter and Si-SPAD. Several previous researches have paved the way for the realization of USPD device. For the up-converter, the internal quantum efficiency (IQE) of 76% with an optimized GaAs LED at room temperature was achieved^24^. Recently, the IQE of InGaAs p-i-n PD can reach about 100% with an extremely low dark current level (~fA)^25^. Using this special designed InGaAs PD, a much higher IQE (>95%) may be realized for the PD-LED up-converter. As for the optical coupling, a contact mask-aligner can be used to press the Si-SPAD device against the up-converter surface during the bonding. And an adhesive thickness of less than 1 μm has been achieved using this technique^37^. The only problem may be the electrode design of the up-converter and Si-SPAD. Different operating modes of the up-converter and Si-SPAD require two independent circuits to control each of them, which need more investigation in the further.

It should be note that the USPD we proposed was based on the inorganic III-V compound semiconductor material. Promoting prototype of the USPD in a much wider range, the semiconductor based PD-LED up-converter can also be replaced by organic PD-LED up-converter^23^ or up-conversion light-emitting phototransistor^38^, which provide greater flexibility for the design of USPD device.

## Conclusion

In conclusion, we proposed a novel single photon detector (USPD) working in free-running regime at the wavelength of 1550 nm based on a relatively mature near infrared up-conversion technology. The structure of USPD consists of an up-converter and a Si SPAD, which are integrated together in three possible ways and controlled by independent circuit. The photons are absorbed in the up-converter and the photon generated carrier multiplication occurs in the Si SPAD. Such unique design makes it possible to suppress the dark count rate and afterpulsing effect efficiently. The PDE, noise, NEP and D^*^ of the USPD are systematically investigated. We found that if the USPD device was fabricated using optical adhesive, the PDE would reach as high as ~45%. Furthermore, the noise performance of the USPD was evaluated and the NEP of an ideal USPD was given as $1.39\times {10}^{-18}{{\rm{WHz}}}^{1/2}$ at 200 K, which is comparable to the theoretical limit of InGaAs SPAD. Finally, a comprehensive evaluation in terms of PDE and NEP among state-of-the-art single photon detectors at the communication wavelength was presented. It was shown that if the up-converter and the Si SPAD are efficiently coupled by optical adhesive, the USPD could acquire a high PDE and a low NEP simultaneously in free-running regime. Meanwhile, the dark count rate and afterpulsing effect can be suppressed to the same level as that of Si SPAD. This means the USPD is a potential excellent single photon detector at 1550 nm wavelength.
